# Cross-View Measurement of Adjacent Fastener Bolt Spacing in Railway Turnouts Using Dual DLP Sensors Without Overlapping Fields of View

**DOI:** 10.3390/s26123943

**Published:** 2026-06-21

**Authors:** Yuntao Gou, Le Wang, Zhixiong Hou, Huchao Zhai, Zichen Gu, Qiyong Wu, Hao Wang, Ning Wang, Qiang Han, Fadeng Wang

**Affiliations:** 1 Infrastructure Inspection Research Institute, China Academy of Railway Sciences Corporation Limited, Beijing 100081, China; 2 Beijing INMAI Railway Technology Co., Ltd., Beijing 100081, China; 3 Guangdong Intercity Railway Operation Co., Ltd., Guangzhou 573100, China

**Keywords:** DLP sensors, non-overlapping fields of view, bridge-based calibration, result validity assessment, railway turnout, bolt spacing measurement

## Abstract

To measure the cross-view spacing between adjacent fastener bolts in railway turnouts, this study develops a dual-DLP-sensor structured-light measurement system without overlapping fields of view. A bridge-type calibration device is used to rapidly update the extrinsic parameters of the two DLP sensors. In a unified coordinate frame, the system integrates two-dimensional region-of-interest candidate generation, local three-dimensional geometric fitting, cross-view pairing, and measurement validity assessment to output bolt-spacing results. Experiments were conducted on 23 pairs of adjacent bolts with 15 repeated measurements using two DLP sensors. Under normal conditions, the mean absolute error, root mean square error, and average standard deviation were 0.261 mm, 0.290 mm, and 0.062 mm, respectively. Compared with fixed extrinsic parameters without updating, the bridge-based extrinsic update reduced the mean absolute error from 1.500 mm to 0.261 mm. The results indicate that the proposed task-driven dual-DLP-sensor measurement system can achieve stable cross-view spacing measurement with explicit validity criteria under non-overlapping fields of view, repeated deployment, and varying on-site data quality.

## 1. Introduction

With the continuous expansion of high-speed railway networks, many existing turnouts have entered long service periods. Recent railway vehicle structural optimization and 3D vision monitoring studies have shown that high-precision geometric data can support railway transit structural assessment, operation and maintenance decision-making, and the construction of digital monitoring frameworks [1,2]. This further highlights the significance of geometric measurement of turnout bolt spacing for railway infrastructure assessment, operation and maintenance decision-making, and digital monitoring frameworks. Turnout construction and maintenance increasingly require rapid acquisition of key assembly parameters. During grouped replacement operations, the available maintenance window is limited, and multiple geometric parameters must be measured and verified within a short time. The spacing between adjacent fastener bolts is one of the key indicators for assembly positioning and construction quality control [3,4,5,6,7,8]. Conventional steel rulers, calipers, and gauges depend strongly on manual operation and are susceptible to reading errors under occlusion, vibration, and restricted pose conditions. Vision- and point-cloud-based automatic measurement can improve efficiency and consistency. However, railway turnout scenes are constrained by installation space, working distance, and local point-cloud density [3,4,9,10,11,12], and multiple DLP sensors are often required for segmented coverage. When two DLP sensors have no overlapping field of view, conventional feature matching or iterative closest point registration lacks common regions and cannot directly establish cross-view coordinate relationships [13,14,15,16,17]. Therefore, cross-view bolt spacing measurement under non-overlapping dual fields of view remains a problem to be addressed.

Existing studies can be broadly divided into three categories. Contact gauges are low in cost but highly operator dependent and provide limited process-quality information. Single DLP sensor or binocular two-dimensional measurements are suitable for detection and local estimation, but their extension to three-dimensional measurement typically relies on overlapping views [6,7,8]. Recent studies on lightweight rail-fastener detection, fastener screw localization, and inspection quantification further improve the robustness and deployability of image-based candidate generation [18,19,20]. Structured-light and multi-sensor point-cloud systems can provide dense local three-dimensional information and are suitable for bolt-top geometric fitting and center extraction [3,4,5,9,10,11,12]. Recent optical 3D reconstruction, pixelwise structured-light calibration, and artifact-based performance evaluation studies also provide useful metrological context for high-precision structured-light measurement [21,22,23]. Nevertheless, coordinate unification under segmented coverage remains necessary.

Non-overlapping multi-DLP-sensor calibration has been studied using rigidly connected targets and sparse three-dimensional maps [13,14,15,16,17], but these methods have not been directly oriented toward cross-view bolt spacing measurement in railway turnouts. Existing work remains insufficient in terms of direct cross-view center-distance output, extrinsic-parameter updating under repeated deployment, and task-coupled result validity assessment. For railway-turnout cross-view fastener-bolt spacing measurement, the system should simultaneously provide sub-millimeter accuracy, rapid extrinsic updating, segmented coverage, and validity assessment, while avoiding dependence on expensive external reference equipment.

This study develops a dual-DLP-sensor structured-light measurement system for cross-view bolt spacing measurement under non-overlapping fields of view. DLP sensors project encoded structured light using a programmable micromirror projection unit and acquire deformed patterns to reconstruct three-dimensional point clouds. Compared with conventional structured-light sensors, DLP sensors offer rapid pattern switching, high stability, and fine grayscale control, making them suitable for dense industrial three-dimensional measurement. The novelty of this study does not lie in proposing a new general theory for non-overlapping DLP sensor calibration. Instead, for the specific metrological task of adjacent railway-turnout fastener-bolt spacing, this work organizes non-overlapping dual-DLP segmented sensing, bridge-based rapid extrinsic updating, local three-dimensional center fitting, cross-view pairing, and result validity assessment into a task-level sensing and measurement chain. This design enables the system not only to unify the coordinates of two views but also to directly output cross-view geometric quantities with quality criteria.

The main contributions are as follows. First, a non-overlapping dual-DLP-sensor segmented-coverage architecture is designed for adjacent fastener-bolt measurement in railway turnouts. Under restricted installation space and limited working distance, the architecture avoids the reduction in point-cloud density caused by a single large field of view and maintains local point-cloud quality on the bolt tops. Second, a bridge-type calibration device and extrinsic update procedure are developed for rapid on-site deployment. The device adopts two spatially separated arrays of reference points on a common rigid body, enabling the two non-overlapping DLP sensors to observe geometric constraints in the same rigid coordinate frame and to recover the cross-view coordinate relationship after transportation, disassembly, and reassembly. Third, a cross-view pairing strategy based on the prior arrangement of railway fasteners is proposed. The strategy combines ordering along the track direction, adjacent-candidate constraints, a distance prior window, and a lateral-offset constraint to reduce mismatching among multiple bolt candidates. Fourth, a task-level validity assessment mechanism is established for the final spacing result. Detection quality, point-cloud quality, fitting quality, pairing consistency, and calibration residuals are mapped to result credibility so that the system can distinguish between valid output, reacquisition, manual review, and recalibration states.

## 2. Materials and Methods

### 2.1. Symbols and Coordinate Definitions

To avoid ambiguity, the notation used in this paper is summarized in Table 1.

The global measurement frame $\left\{G\right\}$ is defined as a physical coordinate frame aligned with the sleeper and track direction. The upper surface of the sleeper is used as the reference plane. The *Z_G_* axis is taken as the normal of this plane, the *Y_G_* axis is aligned with the rail extension direction, and the *X_G_* axis is determined by the right-hand rule. In implementation, *G* does not rely on additional high-precision external equipment. It is estimated from stable geometric structures in the $\left\{S_{1}\right\}$ point cloud. Let ${\hat{z}}_{G}^{S_{1}}$ be the estimated normal of the background plane and ${\hat{y}}_{G}^{S_{1}}$ be the estimated principal track direction. Then ${\hat{x}}_{G}^{S_{1}}={\hat{y}}_{G}^{S_{1}}\times {\hat{z}}_{G}^{S_{1}}$. The rotation $R_{G\leftarrow S_{1}}$ is constructed accordingly. Combined with the bridge-calibrated $R_{S_{1}\leftarrow S_{2}}$ and $t_{S_{1}\leftarrow S_{2}}$, data from the two views can be transformed into $\left\{G\right\}$.

### 2.2. System Composition, Data Acquisition, and Measurement Overview

This section introduces the hardware composition, data forms, and measurement overview of the dual DLP sensor measurement system, and clarifies the input and processing order of the subsequent algorithm chain.

#### 2.2.1. Hardware Composition

The system consists of two VRH9-280B DLP sensors (Imalligent Technology (Shanghai) Co., Ltd., Shanghai, China), a sensor bracket, a bridge-type calibration device, a trigger and acquisition module, and host-computer software (Version 1.0, in-house developed). The two DLP sensors are arranged along the track direction and respectively cover the bolt regions at both ends of the adjacent fasteners. The lateral distance between the two DLP sensors is 600 mm, and their fields of view do not overlap. Each DLP sensor synchronously outputs a two-dimensional texture image and a three-dimensional point cloud with pixel-level alignment.

In this study, the term DLP sensor refers specifically to the commercial VRH9-280B structured-light three-dimensional measurement sensor. The three-dimensional point-cloud reconstruction from Gray-code and phase-shifting fringe images is completed internally by the commercial sensor, and the proposed method uses the output texture images and aligned point clouds for subsequent ROI extraction, geometric fitting, cross-view pairing, and validity assessment. According to the sensor specification, the working-distance range is 450–550 mm; the field of view is approximately 258 × 131 mm, 278 × 145 mm, and 306 × 151 mm at working distances of 450 mm, 500 mm, and 550 mm, respectively; the maximum scanning volume is approximately 278 × 145 × 100 mm; the nominal XY coordinate accuracy is 66 µm; and the nominal Z-axis repeatability is 7.4 µm.

A dual-DLP-sensor segmented-coverage scheme is adopted instead of a single large-field-of-view DLP sensor because an excessively large field of view reduces the effective point-cloud density on the bolt tops, while on-site installation space and viewing angle are constrained by trackside structures. Segmented coverage is more favorable for maintaining a stable working distance and imaging quality.

**Figure 1:**
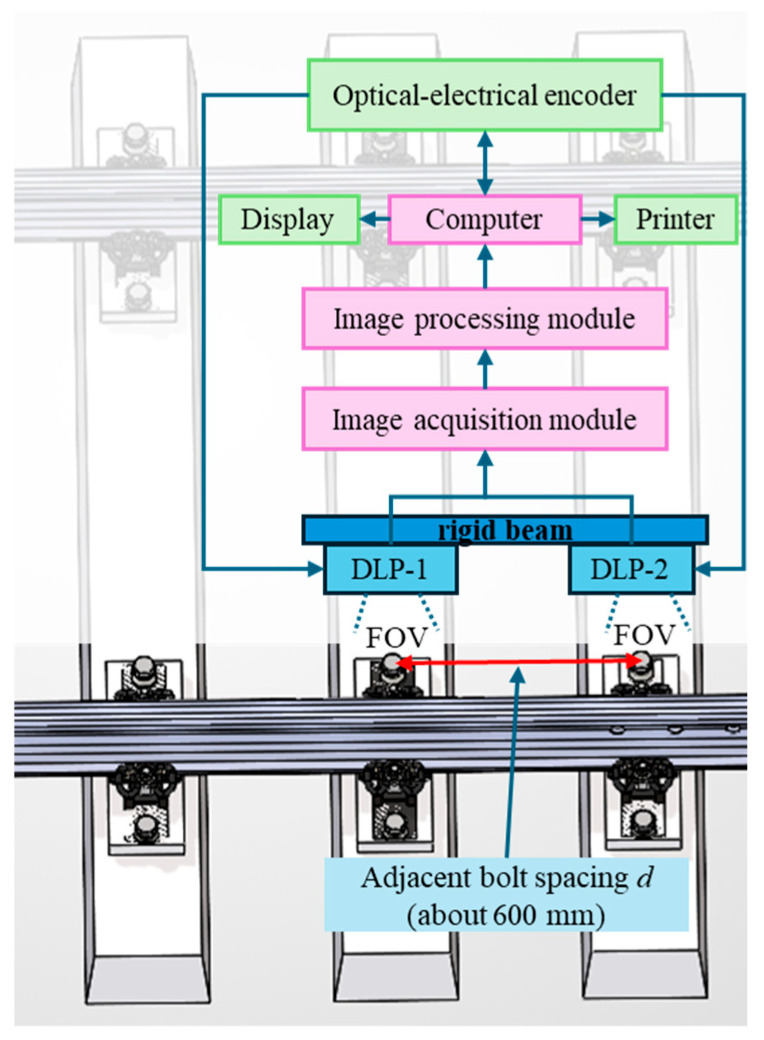
Overall architecture of the dual-DLP-sensor system without overlapping fields of view.

**Figure 2:**
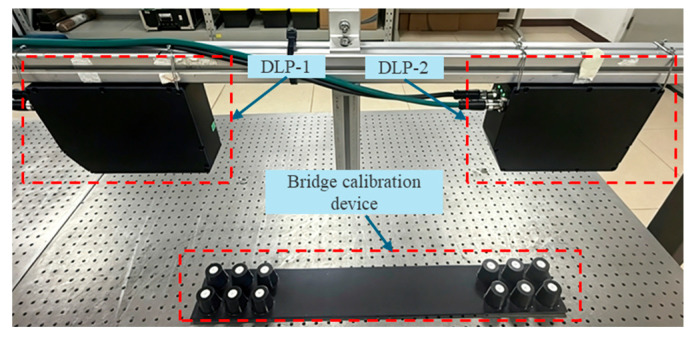
Schematic of the experimental system deployment.

As shown in Figure 1, the system consists of dual-DLP sensing units, a bridge-type calibration device, a trigger and acquisition module, and a host computer; Figure 2 shows the experimental deployment schematic.

#### 2.2.2. DLP Data Types and Pixel Alignment

Each DLP sensor can be regarded as a camera-projector stereo vision system. By projecting Gray-code and phase-shift fringe sequences, the system establishes the corresponding projection ray for each DLP sensor pixel and reconstructs three-dimensional coordinates through triangulation. The key relationship used in this study is

(1) $$\left(u,v)↔I(u,v)↔X^{S_{i}}(u,v\right)$$

The same pixel position corresponds to both the two-dimensional texture intensity and the three-dimensional coordinate. Therefore, candidate regions can first be located in the two-dimensional image, after which geometric fitting can be performed on the aligned point-cloud subset.

#### 2.2.3. Measurement Overview

The complete measurement procedure consists of the following seven steps.

1. Device deployment: install the two DLP sensors and adjust the working distance and viewing angles.
2. Bridge calibration and coordinate unification: place the bridge device, extract reference points on both sides, update extrinsic parameters, and verify residuals.
3. Data acquisition: acquire texture images and point clouds of the fastener regions on both sides.
4. ROI candidate generation and point-cloud preprocessing: locate bolt candidate regions in the two-dimensional texture image and extract and filter the corresponding point-cloud subsets.
5. Bolt center estimation: filter top points and perform local geometric fitting to obtain the bolt centers.
6. Cross-view pairing and distance calculation: complete candidate pairing in the unified coordinate frame and calculate center spacing.
7. Result validity assessment: comprehensively evaluate ROI quality, point count, fitting residual, pairing result, and calibration residual, and output a valid result or a reacquisition recommendation.

The following sections describe bridge-based extrinsic calibration, center estimation, cross-view pairing, and validity assessment in detail.

### 2.3. Bridge-Based Extrinsic Calibration and Coordinate Unification

For the non-overlapping dual-view condition, this section presents the bridge-based extrinsic calibration method and explains the coordinate unification of the two-side measurement results.

#### 2.3.1. Bridge Device and Problem Definition

For two adjacent bolts *a* and *b* located in different fields of view, their centers are $p_{a}^{S_{1}}$ and $p_{b}^{S_{2}}$. Without the relative pose between the two DLP sensors, their cross-view spacing cannot be directly calculated. Therefore, a bridge-type calibration device is introduced to provide external rigid-body geometric constraints, transforming coordinate unification under non-overlapping views into two rigid registration problems.

The calibration module takes as input the three-dimensional reference-point centers observed by the two DLP sensors and the geometric ground truth of the bridge device in $\left\{W\right\}$. It outputs the relative pose $R_{S_{1}\leftarrow S_{2}}$, $t_{S_{1}\leftarrow S_{2}}$ and calibration-quality metrics, including single-DLP-sensor registration RMSE and maximum residual.

The bridge device is designed to satisfy solvability, extractability, repeatability, and verifiability under system constraints. Similar ideas using rigidly connected targets and external constraints have been validated in non-overlapping multi-DLP-sensor calibration studies [14,16,17]. This study applies the idea to railway-turnout cross-view adjacent-bolt spacing measurement.

**Figure 3:**
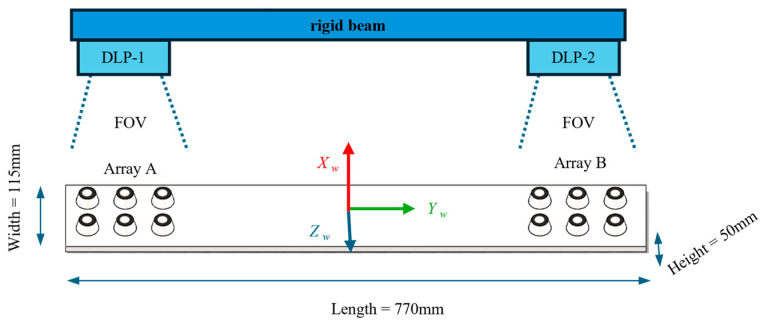
Three-dimensional structure of the bridge calibration device.

As shown in Figure 3, the bridge device consists of two spatially separated arrays, A and B, mounted on the same base plate. The base plate dimensions are 115 mm × 770 mm × 50 mm. The centers of arrays A and B are located at −300 mm and +300 mm along the $Y_{W}$ direction and fall into the fields of view of DLP-1 and DLP-2, respectively. Each array contains six reference points, providing sufficient lateral and longitudinal distribution to ensure observability and redundancy in rigid registration. Each reference point is a truncated-cone protrusion with a circular texture, balancing texture localization and point-cloud geometric fitting. An anti-reflective surface coating reduces instability caused by specular reflection.

#### 2.3.2. Calibration Procedure and Mathematical Model

**Figure 4:**
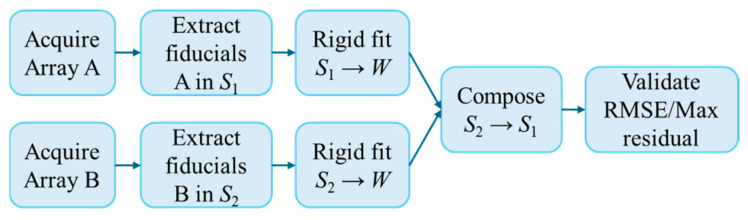
Bridge-based extrinsic calibration procedure.

The bridge-based calibration procedure consists of acquisition, extraction, registration, composition, and validation, as shown in Figure 4.

The texture images and point clouds of arrays A and B are first acquired. The three-dimensional centers of the reference points in each view are then extracted using a 2D localization -> point-cloud filtering -> local fitting procedure. Rigid transformations from $\left\{S_{1}\right\}$ to $\left\{W\right\}$ and from $\left\{S_{2}\right\}$ to {*W*} are solved separately. For sensor i, the rigid registration can be written as

(2) $$\min_{R_{W}\leftarrow S_{i},t_{W}\leftarrow S_{i}}\sum_{k}{\left\|X_{k}^{W}-\left(R_{W\leftarrow S_{i}}X_{ik}^{S_{i}}+t_{W\leftarrow S_{i}}\right)\right\|}_{2}^{2}$$

This problem can be solved in closed form using the singular-value-decomposition-based Kabsch method. From the two registration results, the relative pose between the two DLP sensors is obtained as

(3) $$R_{S_{1}\leftarrow S_{2}}=R_{S_{1}\leftarrow W}R_{W\leftarrow S_{2}}$$

(4) $$t_{S_{1}\leftarrow S_{2}}=t_{S_{1}\leftarrow W}+R_{S_{1}\leftarrow W}t_{W\leftarrow S_{2}}$$

(5) $$R_{S_{1}\leftarrow W}=R_{W\leftarrow S_{1}}^{⊤},t_{S_{1}\leftarrow W}=-R_{W\leftarrow S_{1}}^{⊤}t_{W\leftarrow S_{1}}$$

Finally, the single-DLP-sensor registration RMSE and maximum residual are calculated as calibration-quality criteria:

(6) $${RMSE}_{i}=\sqrt{\frac{1}{K_{i}}\sum_{k}{\left\|X_{k}^{W}-\left(R_{W\leftarrow S_{i}}X_{ik}^{S_{i}}+t_{W\leftarrow S_{i}}\right)\right\|}_{2}^{2}}$$

If either the RMSE or the maximum residual exceeds the threshold, the calibration is judged invalid, and the system prompts reacquisition, outlier removal, or redeployment.

### 2.4. Bolt Center Estimation, Cross-View Pairing, and Validity Assessment

This section describes the complete measurement chain from ROI generation and three-dimensional center fitting to cross-view pairing and result judgment, and provides the distance output and its validity assessment method.

#### 2.4.1. ROI Candidate Generation and Point-Cloud Preprocessing

A two-dimensional rapid candidate generation and three-dimensional precise center estimation chain is adopted. The inputs are the synchronized texture image I(u,v) and point cloud $X^{S_{i}}(u,v)$, and the outputs are the point-cloud subset P of each bolt and its quality label.

To improve robustness under complex background and local occlusion, a lightweight object-detection network is used to generate ROIs. In the experiments, YOLOv8n was selected. The input resolution was 640 × 640, and the network was initialized with pretrained weights and fine-tuned on the dataset collected in this study. During inference, the confidence threshold and non-maximum suppression threshold were set to 0.25 and 0.50, respectively. The training data covered normal, strong-reflection, low-light, occlusion, oil-stain, and other conditions, with 1200 annotated images and 9600 bolt instances. The data were divided into training, validation, and test sets at a ratio of 8:1:1, providing an independent statistical basis for the ROI metrics reported in the results.

For each ROI, the corresponding point-cloud subset P is collected using the pixel-level alignment relationship. If the ROI confidence is insufficient or $|P|<N_{min}$, the sample is labeled as low quality and triggers the ROI validity criterion. $N_{min}$ was set to 800.

Before three-dimensional fitting, point-cloud preprocessing sequentially performs statistical filtering (K = 30, Std = 1.0), voxel downsampling (0.3 mm), and RANSAC plane segmentation (0.5 mm) to remove outliers and the background plane.

#### 2.4.2. Three-Dimensional Geometric Fitting of Bolt Centers

Bolt center estimation is the key step determining final spacing accuracy. Considering that the local bolt top is approximately planar and that its boundary projection is approximately circular or elliptical, a top-point filtering -> local plane fitting -> two-dimensional ellipse fitting -> three-dimensional back-projection strategy is adopted.

**Figure 5:**
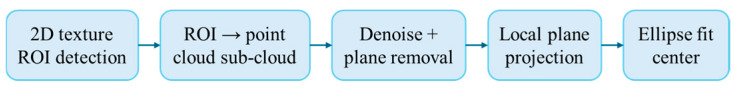
Bolt center extraction procedure.

**Figure 6:**
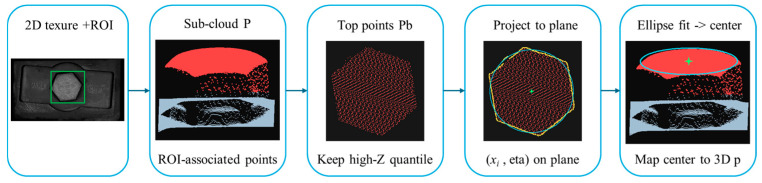
Geometric relationship from 2D ROI to 3D center fitting.

Figure 5 presents the bolt-center extraction procedure, and Figure 6 illustrates the geometric relationship from 2D ROI to 3D center fitting.

Given the point-cloud subset P corresponding to an ROI, the bolt-top point set $P_{b}$ is first obtained through height-quantile filtering:

(7) $$P_{b}=\{x\in P|z(x)\geq z_{p}\}$$

where $z_{p}$ is the p-th quantile of the Z coordinate, with p=0.85, and $\left|P_{b}\right|$ is required to be at least 4000.

A plane is then fitted to $P_{b}$ to obtain the local tangent plane *Π*. The point set is projected onto the plane coordinate system ξ-η. A two-dimensional ellipse is fitted to obtain the center (ξ_0_, η_0_), which is back-projected to the three-dimensional coordinate frame to obtain the bolt center $p^{S_{i}}$. To suppress the influence of outliers, the fitting process can be combined with RANSAC or a robust loss function. The RMS fitting-residual threshold is set to 0.20 mm; samples exceeding this threshold trigger the fitting-validity criterion.

#### 2.4.3. Cross-View Pairing and Distance Calculation

After bolt centers are obtained from the two DLP sensors, they are transformed into the global coordinate frame $\left\{G\right\}$ for pairing. Let $p_{a}^{S_{1}}$ be the bolt center in DLP-1 and pbS2 be the bolt center in DLP-2. Their global coordinates are

(8) $$p_{a}^{G}=R_{G\leftarrow S_{1}}p_{a}^{S_{1}}+t_{G\leftarrow S_{1}}$$

(9) $$p_{b}^{G}=R_{G\leftarrow S_{1}}\left(R_{S_{1}\leftarrow S_{2}}p_{b}^{S_{2}}+t_{S_{1}\leftarrow S_{2}}\right)+t_{G\leftarrow S_{1}}$$

The cross-view distance is defined as

(10) $$d_{ab}={\left\|p_{a}^{G}-p_{b}^{G}\right\|}_{2}$$

During pairing, candidates on both sides are first sorted by their $Y_{G}$ coordinates and then matched within a prior distance window. To reduce mismatching risk, the following constraints are used: (1) distance constraint: $d_{ab}$ in [550, 650] mm; (2) lateral constraint: $|x_{a}^{G}-x_{b}^{G}|\leq 70$ mm; and (3) neighborhood constraint: only the nearest k = 2 candidates after sorting are considered.

Among the candidates satisfying these constraints, the final pairing is determined by minimum distance residual or one-to-one minimum cost. Candidate conflicts, many-to-one matches, or out-of-window results trigger the pairing-validity criterion. The pairing constraints are not arbitrary: the distance window [550, 650] mm is derived from the geometric prior range of adjacent bolt spacing, the 70 mm lateral constraint limits nonphysical cross-view offsets, and k = 2 balances matching flexibility and suppression of long-range mismatching. These parameters were determined from target geometric priors and the mismatch distribution in preliminary experiments.

#### 2.4.4. Measurement Validity Assessment

**Figure 7:**
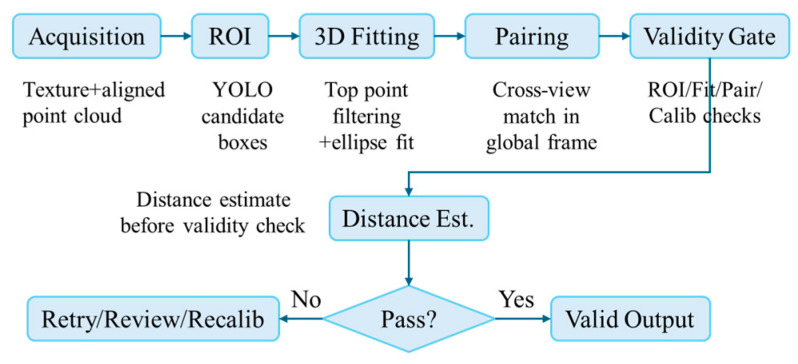
End-to-end measurement chain and validity-decision diagram.

For railway-turnout measurement, the system should output not only distance values but also result credibility and subsequent handling recommendations. Figure 7 shows the end-to-end measurement chain and its validity-decision process. After ROI candidate generation, center estimation, and cross-view pairing, the system comprehensively evaluates four criteria: Validity-ROI, Validity-Fit, Validity-Pair, and Validity-Calib. If any criterion is triggered, the process enters reacquisition, manual review, or recalibration rather than directly outputting the measurement.

Accordingly, the measurement validity assessment module includes four criteria:

1. Validity-ROI: low ROI confidence or insufficient ROI point count.
2. Validity-Fit: insufficient top points or excessive fitting residual.
3. Validity-Pair: pairing conflict, many-to-one pairing, or result outside the structural prior window.
4. Validity-Calib: excessive bridge-calibration residual and invalid extrinsic parameters.

### 2.5. Experimental Design and Evaluation Methods

This section describes the experimental setup, ground-truth acquisition, and evaluation metrics used to verify the accuracy, stability, and robustness of the proposed system and method.

#### 2.5.1. Experimental Design and Data Settings

The experiments address four questions: (1) baseline accuracy and repeatability; (2) the necessity of extrinsic updating under transportation, disassembly, and reassembly; (3) the performance advantage of three-dimensional fitting over two-dimensional center conversion when extrinsic parameters have been updated; and (4) robustness and validity-triggering behavior under strong reflection, low light, occlusion, oil stain, point-cloud holes, and mild vibration.

The proposed method is designed for static acquisition. During data collection, the measurement platform remains stationary, and the two DLP sensors are triggered only after the platform or support has been stably positioned. This assumption is consistent with grouped turnout replacement and acceptance scenarios, in which train operation is suspended, turnout components are installed, adjusted, and checked under maintenance conditions, and static measurement can be performed before traffic resumes. Therefore, dynamic scanning speed, motion blur caused by vehicle running, and synchronization jitter under moving acquisition are outside the target scope of the present study.

The baseline accuracy and repeatability experiment was conducted on 23 adjacent bolt pairs from the same-day static measurement scenario. The bolt pairs corresponded to different adjacent positions within the target turnout area, rather than repeated measurements of a single pair. Different operators participated in the deployment and acquisition procedure, so the reported repeatability includes both the sensing-algorithm chain and practical operator-related deployment effects within the same-day static acquisition boundary.

**Figure 8:**
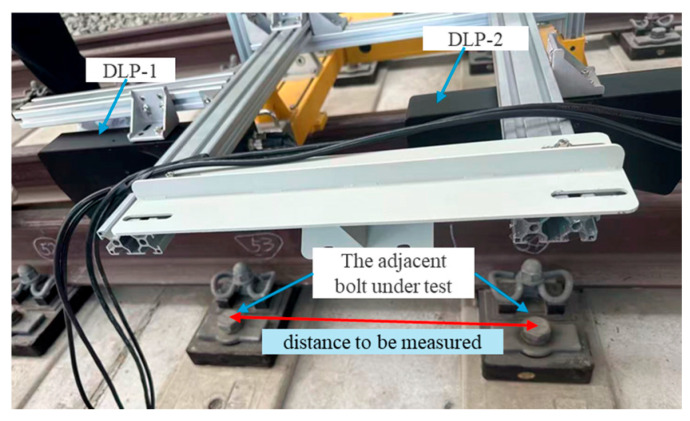
Experimental device for railway-turnout bolt spacing measurement.

The experimental platform used two VRH9-280B DLP sensors, with an XY resolution of 66 µm and a Z repeatability of 7.4 µm. The two DLP sensors were arranged along the track direction with an installation spacing of approximately 600 mm, and their fields of view did not overlap, as shown in Figure 8. Each DLP sensor synchronously output a texture image and a point cloud. The ground-truth distances between adjacent bolts were measured using a calibrated coordinate measuring machine (CMM).

Table 2 summarizes the sample size and statistical scope for each condition. Table 3 summarizes the key thresholds and prior parameters. These parameters were determined according to geometric priors, fitting stability, and calibration/detection performance rather than purely empirical tuning.

Typical data were selected to evaluate the efficiency of the proposed method. The computer CPU was an Intel Core i7-10700K at 3.80 GHz, and the implementation language was C++. Table 4 lists the running time.

#### 2.5.2. Ground Truth and Evaluation Metrics

For the *m*-th pair of bolts, the repeated measurement results are denoted as $\left\{d_{m}^{\left(t\right)}{\}}_{t=1}^{n}\right.$. Their mean and standard deviation are ${\bar{d}}_{m}$ and $s_{m}$, respectively, and the ground truth is $d_{m}^{\backslash }*$. The error is defined as

(11) $$e_{m}={d_{m}^{\backslash }*-\bar{d}}_{m}$$

(12) $$MAE=\frac{1}{M}\sum_{m=1}^{M}|e_{m}|$$

(13) $$RMSE=\sqrt{\frac{1}{M}\sum_{m=1}^{M}e_{m}^{2}}$$

(14) $$e_{max}={max}_{m}|e_{m}|$$

Repeatability is represented by the mean standard deviation $\bar{s}=\frac{1}{M}\sum_{m}s_{m}$. Measurement validity is defined by two metrics: $r_{trig}$ the proportion of acquisitions triggering any validity criterion and requiring reacquisition or manual review; and $r_{fail}$, the proportion of cases in which no valid spacing result can be output after at most $N_{retry}=1$ reacquisition.

## 3. Results

### 3.1. Baseline Accuracy and Repeatability

With bridge calibration used to update the extrinsic parameters, the measurement results for 23 pairs of adjacent bolts are shown in Table 5. The overall MAE, RMSE, maximum absolute error, and average standard deviation over 15 repeated measurements were 0.261 mm, 0.290 mm, 0.517 mm, and 0.062 mm, respectively. For a cross-view measurement distance of approximately 600 mm, the system stably controlled the error within the sub-millimeter range and maintained good repeatability.

### 3.2. Error Bias and Uncertainty Sources

The errors of the 23 samples included both positive and negative values, but the mean error was approximately 0.161 mm, indicating a slight overall underestimation trend. According to the error-propagation analysis, this bias may be related to differences in center definition, zero offsets in extrinsic-parameter composition, and the top-point filtering and fitting model. Table 6 summarizes the main uncertainty sources in a semi-quantitative manner. Extrinsic-updating error and geometric-fitting error are likely the factors requiring priority control, whereas point-cloud quality degradation mainly affects repeatability and the proportion of valid outputs. All reported results are uncorrected raw outputs.

### 3.3. Necessity of Extrinsic Updating

**Figure 9:**
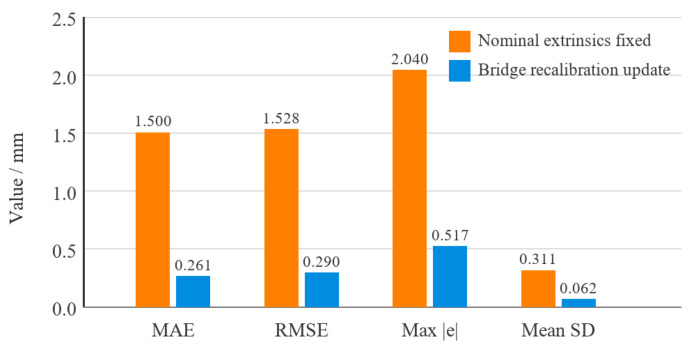
Comparison of summarized metrics between fixed extrinsic parameters and bridge-updated extrinsic parameters.

**Figure 10:**
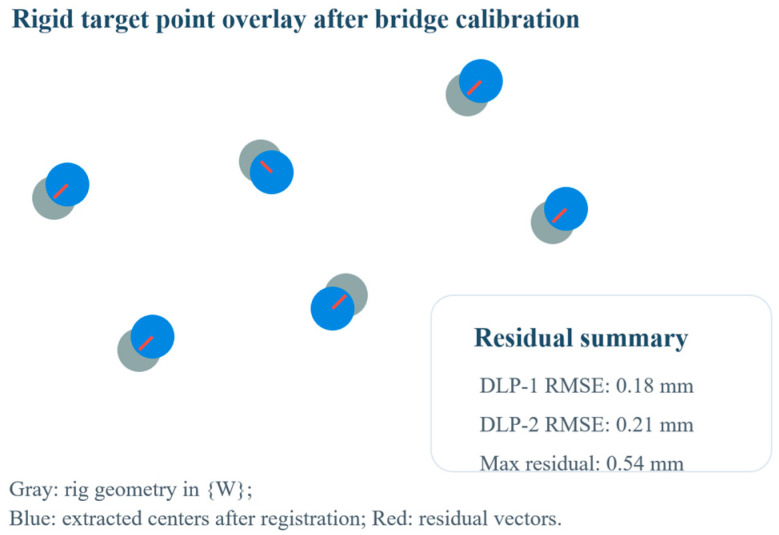
Reference-point registration and residual statistics after bridge calibration.

To verify the necessity of bridge calibration, two strategies were compared: fixed nominal extrinsic parameters without updating, and bridge recalibration before each reassembly. This experiment corresponds to the core issue in non-overlapping multi-DLP-sensor calibration: whether the extrinsic parameters must be explicitly updated under transportation, reassembly, or installation deviations. After six disassembly-transportation-reassembly cycles, the summarized metrics of the two strategies are compared in Figure 9. Fixed extrinsic parameters caused relative-pose drift induced by transportation and clamping to be superimposed as a systematic component on the cross-view distance result, leading to a significantly wider error distribution. Figure 10 shows the reference-point registration and residual statistics after bridge calibration. The results indicate that bridge calibration should be regarded as an extrinsic-refresh step under repeated deployment rather than as a one-time laboratory preparation.

### 3.4. Verification of the Center Estimation Method

To verify the role of the center-extraction model, two center-estimation strategies were compared under updated extrinsic parameters: using only the 2D center, where the ROI-box center was treated as the measurement center and converted using a planar scale, and the proposed 2D ROI + 3D fitting method. The comparison is summarized in Table 7.

The results show that three-dimensional geometric fitting remains essential for reducing measurement error even when extrinsic parameters have been updated. A two-dimensional center cannot explicitly handle changes in bolt-top surface normal, depth fluctuation, or projection distortion. In contrast, three-dimensional fitting provides a more robust parameter estimate through local geometric constraints. This finding is consistent with the conclusion in industrial structured-light measurement that geometric-fitting quality directly affects dimensional-measurement stability.

### 3.5. Robustness and Measurement Validity Under Typical Disturbances

Table 8 lists the accuracy under normal, strong-reflection, and low-light conditions.

The results show that strong reflection and low light increased both error and fluctuation, but the overall accuracy remained within the sub-millimeter range, indicating that the combination of ROI constraints, 3D fitting, and validity criteria provides a degree of tolerance.

For occlusion, oil stain, point-cloud holes, and mild vibration, reporting only MAE is insufficient to characterize system usability. Therefore, the threshold-triggering rate $r_{trig}$ and failure rate $r_{fail}$ were also calculated, as shown in Table 9.

The validity-trigger statistics were further decomposed into four criteria to identify the disturbance source rather than only reporting the aggregated triggering rate and failure rate. The decomposed triggering rates for Validity-ROI/Validity-Fit/Validity-Pair/Validity-Calib were 0.6%/0.7%/0.5%/0.2% under normal conditions, 2.4%/3.1%/1.0%/1.5% under strong reflection, 3.0%/2.2%/0.3%/0.5% under low light, 6.4%/2.6%/4.7%/1.3% under occlusion, 2.0%/6.0%/1.5%/2.5% under oil stain, and 2.2%/3.7%/3.1%/1.0% under mild vibration. This decomposition shows whether a disturbance mainly affects ROI quality, top-surface fitting, cross-view pairing, or bridge-calibration validity, thereby providing a source-localization function in addition to final pass/fail judgment.

**Figure 11:**
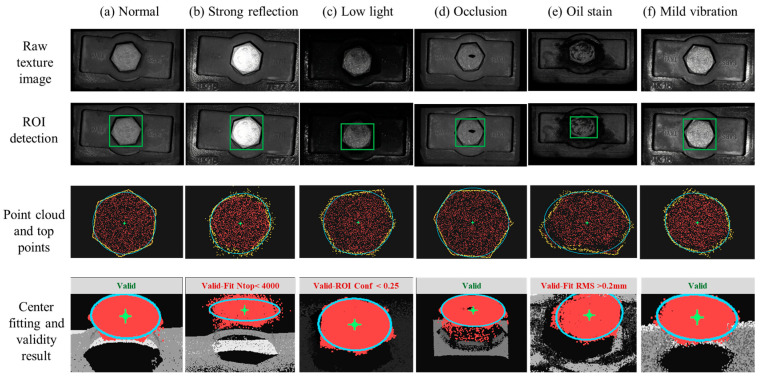
Representative cases of ROI, point cloud, and validity triggering under typical conditions.

Figure 11 shows representative cases of ROI, point cloud, and validity-criterion triggering under typical conditions.

These results indicate that, in addition to final error, it is necessary to identify whether a problem originates from insufficient points, fitting failure, pairing conflict, or calibration abnormality. The validity assessment module can characterize both measurement accuracy and result validity.

Because the ROI detector is responsible for candidate generation, its detection performance under different lighting and reflection conditions was further evaluated to explain the ROI-threshold triggers in Table 9. The evaluation was based on an independent test set of approximately 120 images and 960 bolt instances. The Precision and Recall values in Table 10 were calculated on this test split. For occlusion, oil stain, point-cloud holes, and mild vibration, performance changes were mainly related to point-cloud quality degradation or local geometric-information loss. The ROI module mainly affects the stability of entering the three-dimensional fitting chain, whereas final distance accuracy is still primarily governed by three-dimensional center fitting and extrinsic updating.

## 4. Discussion

### 4.1. Comparison with Baseline Strategies and Manual Measurement

The fixed nominal extrinsic-parameter strategy uses essentially the same hardware as the proposed system but lacks bridge-based extrinsic refreshing and result validity assessment. As shown in Figure 9, the advantage of the proposed system is mainly reflected in reducing the systematic error introduced by reassembly and suppressing erroneous outputs under abnormal inputs through validity assessment. In the experimental platform, a single bridge calibration required approximately 30 s. Considering that transportation and repeated clamping may introduce relative-pose drift, bridge calibration is recommended before each measurement session to update the extrinsic parameters. Compared with manual measurement, the value of the proposed system lies not only in reducing reading dependence but also in forming an automatic measurement chain from sensing acquisition, extrinsic updating, and three-dimensional center estimation to result-credibility judgment. This chain improves the recordability, reviewability, and process traceability of cross-view spacing results.

### 4.2. Comparison with Existing Work

The core contribution of this study is not the replacement of a single algorithmic module but the development of a task-constrained dual structured-light sensing system for railway-turnout cross-view geometric measurement. Unlike studies focusing only on local fastener detection or single-view geometric-parameter measurement, this study directly outputs the center distance between adjacent bolts under non-overlapping fields of view. Unlike general non-overlapping multi-DLP-sensor calibration, this study couples bridge-based extrinsic updating, local three-dimensional center fitting, cross-view pairing based on fastener-arrangement priors, and result-validity judgment to the final spacing task, forming a verifiable end-to-end measurement process.

Compared with railway fastener structured-light inspection studies [3,4,5], this study emphasizes cross-view geometric output and credibility assessment. Compared with industrial structured-light metrology studies [9,10,11,12], it emphasizes extrinsic updating under repeated deployment. Compared with non-overlapping multi-DLP-sensor calibration studies [13,14,15,16,17], it embeds extrinsic recovery into a specific measurement task and result-screening process. Table 11 compares the positioning of this study with related work. The comparison is not intended to demonstrate that a single algorithm outperforms existing methods, but to clarify the integrated differences of the proposed system in terms of task objective, sensing deployment, cross-view output, and result credibility.

Future work will further expand the experimental basis across different dates, more turnout objects, more complex illumination conditions, pollution patterns, reflective surfaces, and longer-term deployment scenarios. Such expanded validation will be used to evaluate long-term generalization beyond the same-day static measurement boundary considered in the present study.

Overall, the main contribution of this study is a system-level sensing design oriented toward railway-turnout measurement. Bridge-based extrinsic updating, local three-dimensional bolt-center fitting, fastener-prior-based cross-view pairing, and result validity assessment are organized into an executable end-to-end chain. The design enables a non-overlapping dual-view system to directly output cross-view bolt spacing with quality criteria, rather than merely completing DLP sensor calibration, local target detection, or single-view geometric measurement. This study has verified baseline accuracy, repeatability, the necessity of extrinsic updating, the contribution of center estimation, and validity-triggering behavior under typical disturbances. However, generalization across dates, operators, and objects still requires further verification; therefore, the current results should be interpreted as performance conclusions within the specified scenario boundaries.

### 4.3. Applicability and Future Extensions

The proposed method is mainly applicable to static or quasi-static bolt-like target measurement. Its prerequisites are stable deployment of the bridge calibration device, relatively consistent working distances and viewing angles of the two DLP sensors, and sufficient bolt-top point-cloud quality for geometric fitting. For continuous vibration, severe occlusion, strong specular saturation, target configurations significantly outside the prior range, or scenes in which the bridge device cannot be stably placed, the current method cannot guarantee the same accuracy and validity-judgment capability. Future work will include long-term stability verification across dates, operators, and objects, as well as uncertainty modeling for abnormal samples, robust reconstruction under complex disturbances, and synchronization compensation for dynamic measurement.

## 5. Conclusions

This study developed a dual-DLP-sensor structured-light measurement system for cross-view spacing measurement of adjacent railway-turnout fastener bolts under non-overlapping fields of view. The system updates the extrinsic parameters of the two DLP sensors through bridge-based calibration and combines two-dimensional ROI candidate generation, local three-dimensional geometric fitting, cross-view pairing, and validity assessment to measure bolt-center spacing in a unified coordinate frame. Under a cross-view distance of approximately 600 mm, the system achieved an MAE of 0.261 mm, an RMSE of 0.290 mm, and an average standard deviation of 0.062 mm under normal conditions. Comparative experiments showed that bridge-based extrinsic updating reduced systematic errors caused by repeated deployment, while three-dimensional geometric fitting improved bolt-center estimation accuracy. The validity assessment module screened low-quality acquisition, fitting abnormalities, pairing conflicts, and calibration abnormalities. The current method is mainly applicable to static or quasi-static scenes and depends on stable bridge-device placement, relatively consistent sensor viewpoints, and bolt-top point-cloud quality satisfying fitting requirements. Further validation is required for continuous vibration, severe occlusion, strong specular saturation, or target configurations that significantly deviate from the prior assumptions.

## Figures and Tables

**Table 1:** Symbols and coordinate conventions.

Symbol	Meaning
$\left\{S_{1}\},\{S_{2}\right\}$	Local coordinate frames of the point clouds output by the two DLP sensors
$\left\{W\right\}$	Rigid coordinate frame of the bridge calibration device
{*G*}	Global measurement coordinate frame aligned with the sleeper/track direction
$R_{A\leftarrow B},t_{A\leftarrow B}$	Rotation and translation that transform points from frame B to frame A
$p_{a},p_{b}$	Center points of two adjacent bolts
$d_{ab}$	Euclidean distance between adjacent bolt centers
$r_{trig}$	Quality-threshold triggering rate
$r_{fail}$	Failure rate under the allowed reacquisition strategy

**Table 2:** Sample size and statistical scope under each condition.

Condition	Bolt Pairs	Repeats	Images	Main Purpose
Normal	23	15	690	Baseline accuracy and repeatability
Strong reflection	10	10	200	Lighting robustness
Low light	10	10	200	Lighting robustness
Occlusion	10	10	200	Failure-mode statistics
Oil stain	6	10	120	Failure-mode statistics
Mild vibration	6	10	120	Failure-mode statistics
ROI test set	-	-	120	ROI Precision/Recall

**Table 3:** Key thresholds and prior parameters.

Module	Parameter	Value	Function
ROI	Confidence threshold	0.25	Triggers ROI threshold when lower than this value
ROI	NMS threshold	0.50	Suppresses duplicated boxes
Preprocessing	Statistical filtering K	30	Outlier removal
Preprocessing	Statistical filtering Std	1.0	Controls filtering strength
Preprocessing	Plane segmentation threshold	0.5 mm	Background-plane removal
Top-point filtering	Quantile threshold *p*	0.85	Extracts top point set
Top-point filtering	Minimum top points	4000	Ensures fitting stability
Fitting	RMS threshold	0.20 mm	Fitting validity judgment
Pairing	Distance prior window	[550, 650] mm	Limits mismatching
Pairing	Nearest candidates k	2	Simplifies matching and suppresses mismatching
Calibration	Single-DLP-sensor RMSE threshold	0.30 mm	Judges extrinsic validity
Calibration	Maximum residual threshold	0.80 mm	Rejects abnormal calibration
Measurement strategy	Maximum reacquisition times	1	Defines the failure-rate scope

**Table 4:** Running-time details.

Module	Time Per Run/s	Description
Bridge calibration	30.00	Two rigid registrations, pose composition, and residual statistics
Data acquisition	1.20	Single structured-light acquisition and decoding
ROI detection	0.03	YOLOv8n inference for a single frame
Point-cloud preprocessing and center fitting	0.18	Statistical filtering, plane segmentation, and ellipse fitting
Pairing and validity judgment	0.02	Prior-constrained matching and threshold checking
Single measurement total (excluding bridge calibration)	1.43	End-to-end processing for one bolt pair

**Table 5:** Measurement statistics for adjacent fastener-bolt spacing.

No.	Ground Truth/mm	Mean of 15 Measurements/mm	Std. Dev./mm	Error/mm
1	631.080	630.955	0.071	0.125
2	598.795	598.939	0.064	−0.144
3	583.785	583.704	0.041	0.081
4	604.415	604.046	0.069	0.369
5	592.465	592.689	0.056	−0.224
6	612.885	612.368	0.085	0.517
7	595.200	595.354	0.046	−0.154
8	600.360	600.102	0.051	0.258
9	613.795	613.639	0.059	0.156
10	589.170	588.770	0.078	0.400
11	607.580	607.641	0.054	−0.061
12	592.420	592.145	0.058	0.275
13	599.730	599.394	0.061	0.336
14	592.595	592.717	0.047	−0.122
15	604.960	604.811	0.049	0.149
16	596.990	596.746	0.053	0.244
17	594.305	593.914	0.075	0.391
18	612.680	612.916	0.081	−0.236
19	597.425	597.108	0.056	0.317
20	581.160	580.874	0.044	0.286
21	616.095	615.644	0.083	0.451
22	604.295	604.502	0.051	−0.207
23	611.880	611.387	0.088	0.493

**Table 6:** Main uncertainty sources.

Source	Influence Mechanism	Level	Main Effect on Spacing Measurement	Suppression Method
Extrinsic-update error	Relative-pose estimation deviation between the two DLP sensors	High	Introduces cross-view systematic bias	Repeated bridge calibration and residual screening
Reference-point geometric error	Bridge-device ground-truth or clamping inconsistency	Medium	Affects extrinsic-solution stability	Rigid design and reference-point maintenance
ROI localization error	Candidate-box offset or missed detection	Medium	Reduces subsequent point-cloud subset quality	Confidence threshold and candidate screening
Point-cloud quality degradation	Reflection, holes, or increased noise	Medium to high	Increases local fitting fluctuation	Preprocessing and validity criteria
Geometric-fitting error	Top-point filtering and ellipse-fitting bias	High	Introduces center-estimation bias	RMS threshold and robust fitting
Pairing error	Cross-view candidate matching conflict	Low frequency, high impact	Produces significant distance anomalies	Distance window, lateral constraint, and one-to-one matching

**Table 7:** Comparison between 2D center only and 2D ROI + 3D fitting.

Method	MAE/mm	RMSE/mm	Maximum Error/mm	Average Std. Dev./mm
2D center only (after bridge calibration)	0.900	0.960	1.650	0.120
Proposed method (2D ROI + 3D fitting)	0.261	0.290	0.517	0.062

**Table 8:** Performance degradation under different lighting conditions.

Condition	MAE/mm	RMSE/mm	Maximum/mm	Average Std. Dev./mm
Normal	0.261	0.290	0.517	0.062
Strong reflection	0.420	0.450	0.750	0.085
Low light	0.380	0.410	0.720	0.080

**Table 9:** Triggering and failure rates under typical failure modes.

Condition	Main Phenomenon	$r_{trig}$	$r_{fail}$	Main Triggered Criteria
Normal	Baseline condition	2.0%	0.0	Fitting residual, ROI point count, pairing conflict
Strong reflection	Strong reflection and local saturation	8.0%	1.0%	Fitting residual, ROI confidence, point-cloud holes
Low light	Low illumination and reduced contrast	6.0%	0.5%	ROI confidence, ROI point count, fitting residual
Occlusion	Local occlusion	15.0%	3.0%	ROI/top-point count and pairing conflict
Oil stain	Oil or water stains	12.0%	2.0%	Fitting residual and point-cloud holes
Mild vibration	Mild vibration and texture blur	10.0%	1.5%	ROI confidence, fitting residual, pairing conflict

**Table 10:** Precision and Recall of the ROI detector under different conditions.

Condition	Precision/%	Recall/%	Typical False or Missed Detection Source
Normal	97.6	97.9	A small number of false detections caused by similar background textures
Strong reflection	95.3	93.2	Missed detections caused by top saturation and boundary disappearance
Low light	96.5	92.1	Missed detections caused by reduced contrast and noisy textures

**Table 11:** Positioning comparison between this study and related work.

Dimension	Railway Fastener Structured-Light Measurement/Detection [3,4,5]	Non-Overlapping Multi-DLP-Sensor Calibration [13,14,15,16,17]	This Study
Main objective	Fastener geometric-parameter measurement, defect detection, or point-cloud recognition	General extrinsic recovery and DLP-sensor-array calibration	Cross-view spacing measurement of adjacent fastener bolts in railway turnouts
Specific application scenario	Partly related, but usually not focused on cross-view adjacent bolts	Usually not focused on specific railway scenes	Yes
Treatment of non-overlapping fields of view	Usually not a core issue	Yes	Yes
Task-coupled final geometric output	Mostly local parameters or detection results	Usually does not directly output railway task quantities	Yes, directly outputs cross-view spacing
Result validity assessment	Usually weak	Usually focuses on calibration accuracy	Yes
Focus	Fastener recognition or local measurement	Calibration accuracy and generality	Extrinsic updating, center fitting, and result validity assessment

## Data Availability

Data underlying the results presented in this paper are not publicly available at this time but may be obtained from the authors upon reasonable request.
